# Survey of 5 mm small‐field output factor measurements in Australia

**DOI:** 10.1002/acm2.12259

**Published:** 2018-01-25

**Authors:** Christopher P. Oliver, Duncan J. Butler, Viliami Takau, Ivan Williams

**Affiliations:** ^1^ Australian Radiation Protection and Nuclear Safety Agency Yallambie Vic Australia

**Keywords:** small‐field dosimetry, output factor, stereotactic cone

## Abstract

The Australian Radiation Protection and Nuclear Safety Agency (ARPANSA) held a comparison exercise in April 2016 where participants came to ARPANSA and measured the output factor of a nominal 5 mm cone attached to the ARPANSA Elekta Synergy (Elekta, Crawley, UK) linear accelerator. The goal of the exercise was to compare the consistency and methods used by independent medical physicists in measuring small‐field output factors. ARPANSA provided a three‐dimensional scanning tank for detector setup and positioning, but the participants were required to measure the output factor with their own detectors. No information regarding output factors previously measured was supplied to participants to make each result as independent as possible. Fifteen groups travelled to ARPANSA bringing a wide range of detectors and methods. A total of 30 measurements of the output factor were made. The standard deviation of the measurements (excluding one expected outlier from an uncorrected ionization chamber measurement) was 3.6%. The results provide an insight into the consistency of small‐field dosimetry being performed in Australia and New Zealand at the present time.

## 1 INTRODUCTION

Reference dosimetry for conventional external beam radiotherapy in Australia is generally performed using the IAEA TRS‐398 Code of Practice which specifies a 10 × 10 cm field in which to measure the absorbed dose to water in the user's megavoltage (MV) photon beam.^1^ Increasingly, smaller MV photon fields are being used for patient treatments which bear little resemblance to the broad field measured during reference dosimetry.

Newer stereotactic treatments are hypofractionated and deliver large doses to localized areas with smaller nonconventional fields. Some new radiotherapy treatment machines are incapable of delivering a 10 × 10 cm field, and thus, the dosimetry must be performed by another method. The reference dosimetry of these small fields is complex and cannot always be performed with conventional equipment. Alfonoso et al. published a small‐field formalism in 2008 which is being widely used and presents the need for new beam‐specific correction factors when measuring small fields.^2^ Small‐field dosimetry can be performed with a variety of detectors and each has associated strengths and weaknesses.^3^ The measurement of correction factors for all detectors in common clinical situations is a large task and currently ongoing. There is presently no accepted protocol to use when performing small‐field dosimetry, although efforts are underway.^2^, ^4^

With the lack of an accepted protocol, it is currently up to the clinical medical physicist to decide how to perform dosimetry of small fields. The choice of detector and use of associated correction factors are very important for dosimetric accuracy. Das et al. described the challenges of small‐field dosimetry and how they could be overcome.^3^ Cranmer‐Sargison et al. measured output ratios for a variety of diodes on two different accelerators highlighting some of the practical aspects of small‐field dosimetry.^5^ Kairn et al. have published a practical guide on how to perform small‐field output factor measurements with diodes and microchambers.^6^

The comparison exercise was initiated to investigate the consistency of small‐field measurements in Australia. ARPANSA does not have a primary standard for absorbed dose in small fields which can measure the dose output of the nominal 5 mm cone. Therefore, the results presented here will not be compared to a primary standard measurement of the output factor. ARPANSA participated in the comparison, but its results should not be considered any more valid or accurate than any other result in the comparison.

## 2 METHOD

**Figure 1:**
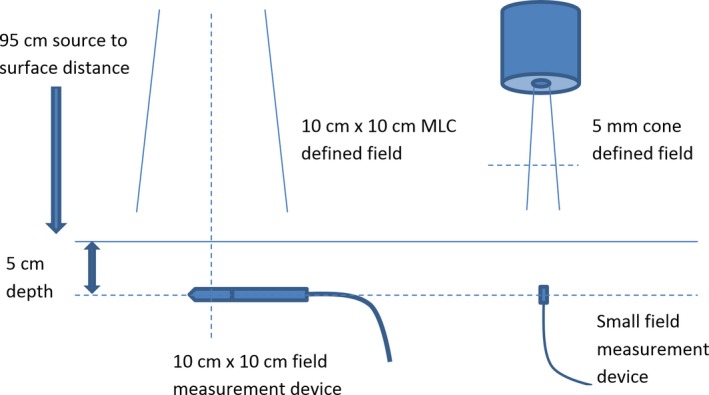
Experimental setup for the small‐field output factor measurement indicating the geometry for the reference and small fields. In this example, the 10 × 10 cm field was being measured by a Farmer chamber and the small field by a diode; however, the detector types were chosen by each participant. The measurement by both detectors in an intermediate field to relate the two displayed measurements is not shown.

Each participant was given detailed information well before their allocated time to allow appropriate choice of detectors for the measurements needed. All measurements were performed on the ARPANSA Elekta Synergy linear accelerator using the 6 MV (*TPR*_*20,10*_ = 0.673) photon beam. The output factor to be measured was the ratio of the dose in the 5 mm cone‐defined field to the dose in the 10 × 10 cm multileaf collimator (Elekta MLCi2)‐defined field for a given number of MU. The reference conditions for the measurement are shown in Fig. 1.

The 5 mm cone was commercially manufactured (Elekta Stereotactic Collimator, product number TRT 0065). It was screwed into a frame (Elekta product number MRT 13541) which was bolted onto the head of the linear accelerator. The frame, when in position, was located approximately 60 cm from the location of the accelerator target. The cone was composed of a combination of Sn, Bi, and Pb. Its length was 9 cm and it produced a circular field at isocenter of nominal diameter 5 mm. It was not interlocked with the accelerator and care was taken to ensure that the jaws were set to a field size of 3 × 3 cm when the cone was in place. This setting was previously determined to inhibit any radiation leaking around the cone and minimize any effect on the radiation passing through the cone. The reproducibility of the cone placement was tested by removing and remounting the cone and frame nine times. The maximum difference in the field produced by the cone in both lateral directions (i.e., perpendicular to the beam direction) was 0.2 mm. This was measured by scanning the field with a PTW 60017 electron diode (PTW‐Freiburg GmbH, Freiburg, Germany) at a source to surface distance (SSD) of 95 cm and depth of 5 cm in inline and crossline orientations to determine the central position of the field. The effect on the output factor of a cone misalignment was measured with a PTW 60017 electron diode and a PTW 60019 microDiamond detector. The cone was deliberately shifted by 0.3 mm in both inline and crossline directions to simulate the worst‐case scenario misalignment after removing and then replacing the cone. If the detector was not realigned with the center of the field, the percentage change in the measured output factor was −1.4% and −0.8% for the microDiamond and electron diode, respectively. It was expected that the larger diameter detector will see the biggest difference, and that is what was observed. However, if the detectors were realigned in the field with the shifted cone, the change in output factor measured for both detectors was less than 0.1%. The dosimetric field width of the 5 mm cone field was defined as the 50% isodose level normalized to central axis. It was measured with a PTW 60017 electron diode and a PTW 60019 microDiamond at 5 cm depth in a water phantom with a 95 cm source to surface distance. Both detectors were oriented parallel to the beam direction. The crossline full width half maximum was 5.8 mm and the inline full width half maximum was 5.9 mm, with both detectors agreeing to within 0.1 mm in both directions.

An isocentric setup of 95 cm SSD and 5 cm depth in water was chosen for the comparison. These values were based on the feedback given by several clinics who had measured small‐field output factors for commissioning data for a range of treatment planning systems. As there is no accepted protocol for small‐field dosimetry and different treatment planning systems have different SSD and depth setups, the choice was arbitrary. The combination of SSD of 95 cm and depth of 5 cm had the advantage that IAEA TRS‐398 could be used to perform reference dosimetry in the 10 × 10 cm field for the 6 MV photon beam.

ARPANSA provided an IBA (IBA Dosimetry GmbH, Schwarzenbruck, Germany) Blue Phantom 3D scanning water tank with associated software, virtual water, adapters, electrometer, triaxial cables, and various mounts for participant detectors in the water phantom. All detectors were brought to ARPANSA and mounted in the water tank as directed by the participant. The order of the experiments and what measurements were made were also directed by the participant. ARPANSA provided no input or suggestion as to how the measurements should take place but aided with the logistics of the measurements such as mounting of detectors without standard holders in the water phantom. The participants were not given any information about previous output factor measurements so as not to influence their results. However, the comparison was not blind in a rigorous sense as cone factors are available in the literature, and discussions between the groups were possible when schedules overlapped.

## 3 RESULTS

The departments that participated in the comparison are listed in alphabetical order in Table 1. Table 2 lists the types and dimensions of the active detectors used in the comparison. All results including some experimental details are shown in Tables 3 and 4. Table 3 shows the active detector results while Table 4 shows Gafchromic EBT3 film results. The “Uncorrected output factor” in Table 3 is the ratio of the small‐field detector reading in the 5 mm field to the small‐field detector reading in the 10 × 10 cm field if it was used in both the fields. Otherwise, it is the ratio of the reading of the small‐field detector in the 5 mm field and an intermediate field multiplied by the ratio of readings of the large‐field detector in the chosen intermediate field and the 10 × 10 cm field. The use of and selection of intermediate field size were left to each participant. These results are presented to show the influence of the subsequent corrections applied and to allow these results to be compared using different correction factors. The correction factors used by the participants came from a variety of sources and these are shown in the table. For the active detectors, each participant was also asked if this detector was used clinically and if so, what was the smallest field measured with this detector. This is also shown in Table 3.

**Table 1:** Participant organizations

Organization
Andrew Love Cancer Centre, Geelong, Vic
ARPANSA, Vic
Calvary Mater Newcastle, Waratah, NSW
Casey Radiation Oncology Centre, Berwick Vic
Chris O'Brien Lifehouse, Camperdown, NSW
Olivia Newton‐John Cancer Wellness & Research Centre, Austin Health, Heidelberg, Vic
Peter MacCallum Cancer Centre, Sunshine, Vic
Prince of Wales Hospital, Randwick, NSW
Radiation Oncology Institute, Wahroonga, NSW
RMIT University, Melbourne, Vic
Royal Adelaide Hospital, Adelaide, SA
The Alfred Hospital, Melbourne, Vic
The Canberra Hospital, Garran, ACT
Townsville Cancer Centre, Douglas, QLD
Vector Lab, University of Sydney, NSW
Waikato Hospital, Hamilton, New Zealand

**Table 2:** Materials and dimensions of the active volumes of the detectors used in the comparison exercise

Detector	Active detector material	Active detector dimensions perpendicular to the beam	Active detector length parallel to the beam	Active volume
IBA Razor detector	Unshielded p‐type silicon diode	0.6 mm diameter	0.02 mm	0.006 mm^3^
Sun nuclear edge detector	Silicon diode encapsulated in brass housing	0.8 × 0.8 mm^2^	0.03 mm	0.019 mm^3^
IBA SFD stereotactic diode	Unshielded p‐type silicon diode	0.6 mm diameter	0.06 mm	0.02 mm^3^
IBA EFD electron diode	Unshielded p‐type silicon diode	2 mm diameter	0.06 mm	0.2 mm^3^
PTW 60019 microDiamond	Diamond Schottky diode	2.2 mm diameter	0.001 mm	0.004 mm^3^
PTW 60018 SRS diode	Unshielded p‐type silicon diode	1.2 mm diameter	0.25 mm	0.3 mm^3^
Aircore plastic scintillation detector	BC400 plastic scintillator	1 mm diameter	1 mm	0.8 cm^3^
PTW 60017 electron diode	Unshielded p‐type silicon diode	1.2 mm diameter	0.03 mm	0.03 mm^3^
Bragg peak chamber	PMMA‐walled ionization chamber vented to air	82 mm diameter	2 mm	10.5 cm^3^
PTW 31016 PinPoint 3D	PMMA‐walled ionization chamber vented to air	2.9 mm diameter	2.9 mm	0.016 cm^3^
PTW 31014 PinPoint	PMMA‐walled ionization chamber vented to air	2 mm diameter	5 mm	0.015 cm^3^
IBA CC04	C552‐walled ionization chamber vented to air	4 mm diameter	3.6 mm	0.04 cm^3^
Standard imaging A1SL	C552‐walled ionization chamber vented to air	4 mm diameter	4.4 mm	0.053 cm^3^

**Table 3:** Summary of results for all active detectors

5 mm field detector	Intermediate field	10 × 10 cm field detector	Uncorrected output factor	Stated uncertainty^a^	Correction factor	Source of correction factor	Output factor	Stated uncertainty^a^	Clinical use	Smallest clinical field
IBA Razor diode	NA	IBA Razor diode	0.637	0.61%	None	NA	0.637	0.61%	No	NA
IBA Razor diode	5 × 5 cm	IBA FC65‐G	0.653	3.02%	0.974	7	0.636	3.62%	Yes	1.5 cm^2^
Sun nuclear edge	NA	Sun Nuclear Edge	0.668	3.74%	0.945	8	0.632	4.17%	Yes	2 × 2 cm
PTW 31016 pinpoint 3D	NA	PTW 31016 Pinpoint 3D	0.529	3.74%	1.12	9	0.592	4.13%	Yes	2 × 2 cm
IBA SFD stereotactic	2 × 2 cm & 5 × 5 cm	IBA CC04	0.628	2.78%	None	NA	0.628	2.78%	Yes	4 mm cone
IBA EFD electron	4 × 4 cm	IBA CC13	0.638	1.60%	0.955	10	0.610	1.68%	Yes	10 mm cone
IBA SFD stereotactic	5 × 5 cm	PTW pinpoint 3D chamber	0.639	1.46%	0.961	11	0.614	2.48%	Yes	1 × 1 cm
PTW 60019 microDiamond	NA	PTW microDiamond	0.643	5.0%	0.969	10	0.623	5.00%	No	NA
IBA RAZOR diode	4 × 4 cm	IBA CC13	0.652	0.10%	None	NA	0.652	0.10%	Yes	5 mm cone
Sun nuclear edge	NA	Sun Nuclear Edge	0.667	1.00%	None	NA	0.667	1.00%	Yes	1.5 × 1.5 cm
PTW 60019 microDiamond	NA	PTW 60019 microDiamond	0.645	1.00%	0.961	10	0.620	1.00%	Yes	1.5 × 1.5 cm
Standard imaging A1SL	NA	Standard Imaging A1SL	0.471	1.00%	None	NA	0.471	1.00%	Yes	2 × 2 cm
PTW T60018 SRS Diode	3 × 3 cm & 5 × 5 cm	IBA CC04	0.658	1.50%	0.929	12	0.612	3.00%	Yes	1 × 1 cm
IBA CC04	NA	IBA CC04	0.500	0.2%	1.141	–b	0.570	3.40%	Yes	1 × 1 cm
IBA EFD electron	3 × 3 cm	IBA CC13	0.623	1.00%	0.974	10 & 13 & 14	0.606	2.90%	No	NA
IBA SFD stereotactic	3 × 3 cm	IBA CC13	0.618	1.00%	0.966	10 & 13 & 14 & 15	0.598	3.00%	No	NA
PTW 34070 Bragg Peak chamber + Gafchromic EBT3 film	5 cm cone field	IBA FC65‐G	NA	NA	NA	NA	0.645	4.90%	No	NA
IBA Razor diode	3 × 3 cm	IBA CC04	0.641	0.16%	1.002	–b	0.642	0.16%	No	NA
Air core plastic scintillator	NA	Air core plastic scintillator	0.599	0.67%	1.007	–b	0.603	0.66%	Yes	4 mm cone
PTW 60017 electron diode	NA	PTW 60017 electron diode	0.632	3.00%	0.939	11	0.594	3.20%	No	NA
PTW 60019 microDiamond	NA	PTW 60019 microDiamond	0.601	3.00%	1.027	16	0.618	3.20%	No	NA
PTW 31014 pinpoint chamber	NA	PTW 31014 pinpoint chamber	0.490	3.20%	1.22	17	0.598	3.5%	No	NA

a Expanded uncertainty (k = 2) defining an interval having a level of confidence of approximately 95% in the output factor (or uncorrected output factor), as stated by the facility. The reasons for the large variations between these values are discussed in the text.

b Volume averaging correction calculated from profile measurements.

**Table 4:** Summary of all results using Gafchromic EBT3 film

Output factor	Stated uncertainty^a^	Linearity curve measured at ARPANSA	MU delivered for 5 mm field irradiations	Number of films irradiated in 5 mm field	MU delivered for 10 × 10 cm field irradiations	Number of films irradiated in 10 × 10 cm field	Size of region of interest for 5 mm cone field	Scanner Brand/Model
0.601	3.35%	No	500	3	500	3	0.35 mm^2^	EPSON (Seiko Epson Corp., Nagano, Japan) 10000XL
0.639	6.00%	No	500	1	500	1	1 × 0.5 mm	EPSON V700 red channel
0.611	0.80%	Yes	200	3	200	3	1.4 × 1.4 mm	EPSON Expression 10000XL
0.610	3.40%	Yes	434	1	Multiple exposures, 200–300 MU	4	4 pixels	EPSON V700
0.570	4.56%	No	154	5	92	5	0.7 × 0.7 mm	EPSON 10000XL
0.615	3.25%	No	285	3	200	3	Zero area extrapolation technique 18	EPSON 10000XL
0.621	4.50%	Yes	400	1	100 MU FC‐65G used to get CAX dose	NA, IBA FC‐65G ionization chamber used	1 mm diameter	EPSON 10000XL red channel
0.599	3.67%	Yes	300	5	300	5	0.7 × 0.7 mm	EPSON 10000XL

a Expanded uncertainty (k = 2) in the output factor (or uncorrected output factor), as stated by the facility.

Table 4 displays the Gafchromic (Ashland Inc., KY, USA) EBT3 film measured output factors and gives some information about the measurements and subsequent analysis. Some groups measured a dose linearity curve at ARPANSA although this was challenging considering the limited time. We believe that they did this to ensure that the same batch of film was used for both output and linearity measurements and to account for any possible energy dependence of the film linearity. The number of Gafchromic EBT3 films irradiated in each field and the monitor units delivered are given. The size of the region of interest used for the dose determination and the scanner used for the Gafchromic EBT3 film readout are also shown.

**Figure 2:**
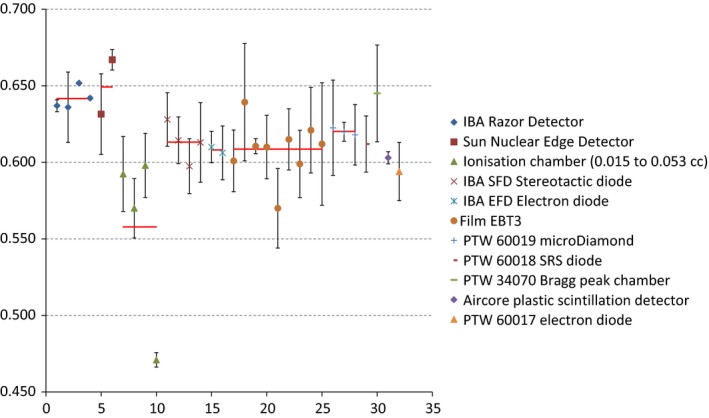
All reported output factors grouped according to detector type with their associated average value shown as a horizontal line.

All reported output factor results shown in Fig. 2 were separated into different detector types. The average of all the output factors measurements was 0.611 or 0.616 excluding the uncorrected ionization chamber measurement which is an obvious outlier. The standard deviation of all reported output factors was 5.6%. If the outlier ionization chamber result is excluded, the standard deviation reduces to 3.6%. The red lines indicate the average value reported for each different type. The displayed error bars for each point are the expanded uncertainties with a confidence level of 95% and were calculated by the participant.

The results for each detector type are summarized below.

### 3.A IBA (IBA Dosimetry GmbH, Schwarzenbruck, Germany) Razor detector

There were four measurements performed with the IBA Razor diode. In one case, the diode was used in the 10 × 10 cm and 5 mm fields; however, in the other three cases, the response of the diode in the 5 mm field was “daisy chained” to the response of an ionization chamber in the 10 × 10 cm field through an intermediate field.^19^ For one result, a correction factor was estimated from Cranmer‐Sargison et al., 7 and for another result, a volume averaging correction was applied calculated using the 5 mm field profile. The remaining two results did not apply a correction factor. The standard deviation of the results was 1.1% and the average value output factor reported with this detector was 0.642.

### 3.B Sun Nuclear (Sun Nuclear, FL, USA) Edge detector

Two measurements were made with the Sun Nuclear Edge detector. In both the cases, the detectors were used in the 5 mm cone fields and 10 × 10 cm fields. In one case, a correction factor was applied from Bassinet et al., 8 and in the other case, no correction was applied. The standard deviation of the two results was 3.9% and the average was 0.649.

### 3.C PTW (PTW‐Freiburg, Freiburg, Germany), IBA, and Standard Imaging (Standard Imaging Inc., WI, USA) Ionization chambers

Four measurements of the output factor were made with ionization chambers. These were type PTW 31016 PinPoint 3D, PTW 31014 PinPoint, IBA CC04, and Standard Imaging A1SL chambers with active volumes ranging from 0.015 to 0.053 cc. Each chamber was used to measure the 5 mm field and the 10 × 10 cm field. No correction was applied to the A1SL result, and it is an obvious outlier being significantly lower than the other ionization chamber reported output factors. The correction factors for the three corrected ionization chamber measurements were large and ranged from 12% to 22%. Two were from the literature^9^, ^17^ and one was based on a volume averaging correction from a measured profile. The average of these four results was 0.558 with a standard deviation of 10.6%.

### 3.D IBA SFD stereotactic

Three measurements were made with the IBA SFD Stereotactic diode. All measurements in the 5 mm field were daisy chained back to ionization chamber measurements in the 10 × 10 cm field via intermediate fields. Correction factors from the literature were applied to two of the results with the other result being uncorrected. The correction factors applied were from a range of sources and one was a weighted average from five different papers.^10^, ^11^, ^13^, ^14^, ^15^ The average of the reported output factors was 0.613 with a standard deviation of 2.5%.

### 3.E IBA EFD electron

There were two instances of output factor measurements with the IBA EFD Electron diode. Both measurements in the 5 mm field were daisy chained back to IBA CC13 measurements in the 10 × 10 cm field through intermediate fields. Correction factors from the literature were applied to both the results with one being a weighted average of correction factors from more than one paper.^10^, ^13^, ^14^ The average of the output factors was 0.608 and the standard deviation 0.4%.

### 3.F Gafchromic EBT3 film (Ashland Inc., KY, USA)

The most popular method of measuring the output factor was with Gafchromic EBT3 film with eight participants using this method. All but one of the film measurements in the 5 mm field was compared to film measurements in the 10 × 10 cm field. The other 5 mm field measurement was compared to an ionization chamber measurement in the 10 × 10 cm field. Four participants measured linearity curves for their film while at ARPANSA, and three participants scaled the MU delivered to obtain similar optical densities for both measured fields. The remaining participants delivered the same MU for both 5 mm and 10 × 10 cm fields. One participant irradiated their films in the water phantom, but all others used Virtual Water™ (Med‐Cal, WI, USA) supplied by ARPANSA. One participant used a new extrapolation technique to obtain their final value.^18^ We do not believe that the use of Virtual Water™ will significantly affect the film‐measured output factors. We have measured percentage depth dose curves in this beam in Virtual Water™ and a water phantom and observed no local differences down to a depth of 20 cm. The average of the output factor measurements made by film was 0.608 with a standard deviation of 3.3%.

### 3.G PTW 60019 microDiamond

Three measurements were made with PTW 60019 microDiamond detectors. In all three cases, the microDiamond was used in both the 5 mm and 10 × 10 cm fields. All results were corrected with data from the literature.^10^, ^16^ Interestingly, the published correction factors do not agree on whether the microDiamond will under respond or over respond in the 5 mm field. The average of the microDiamond output factor results was 0.620 with a standard deviation of 0.4%.

### 3.H PTW 60018 SRS diode

One measurement was performed with a PTW 60018 SRS diode. Its response in the 5 mm field was daisy chained to the response of an IBA CC04 ionization chamber in the 10 × 10 cm chamber through an intermediate field of size 3 × 3 cm. A correction factor from the literature was applied to the result.^12^ The output factor reported was 0.612.

### 3.I PTW 60017 electron diode

One measurement was made with the PTW 60017 electron diode. It was used to measure the output in both the 5 mm and 10 × 10 cm fields. A correction factor from the literature^11^ was applied to obtain the reported output factor value of 0.594.

### 3.J PTW 34070 Bragg peak chamber plus Gafchromic EBT3 film

A technique using a PTW 34070 Bragg peak chamber for measurements of dose area product (DAP)^20^, ^21^ combined with relative dose profiles measured with Gafchromic EBT3 film was also used to derive the output factor. The chamber was first calibrated in a large 50 mm cone field with a known central axis dose rate from secondary standard Farmer chamber measurements. The central axis dose multiplied by the Gafchromic EBT3 film‐determined relative dose profile over the chamber divided by the chamber response gives the chamber calibration factor. The chamber calibration factor multiplied by the chamber reading in the 5 mm field divided by the relative dose profiles over the active area in the 5 mm field gave the central axis dose in absolute terms. The central axis dose was then divided by the central axis dose in the 10 × 10 cm MLC field measured with a secondary standard farmer chamber to obtain the output factor. All Bragg Peak chamber responses were corrected for saturation, polarity, and air density. Relative dose profiles were normalized to central axis and based on a combination of two Gafchromic EBT3 film exposures at high (4000 MU) and low (400 MU) doses, with the aim of increasing the accuracy in the low‐dose region outside the penumbra. The output factor measured with this technique was 0.645.

### 3.K Air core plastic scintillation detector

A noncommercial air core plastic scintillation detector was also used to measure the output factor. It was used in both the 5 mm and 10 × 10 cm fields. No correction factor was applied to the ratio of the readings from each field. The value reported from this detector was 0.603.

## 4 DISCUSSION

This aim of this study was to investigate the consistency of small‐field measurements in Australia, by comparing a small‐field output factor measured at ARPANSA by different physicists. We recognize that most participants had only 2 hours to perform their measurements, and thus, there may have not been enough time to perform the measurements in exactly the same way as would be done in their clinical environment. Based on feedback from the participants, we believe that the results are still broadly representative of what is achieved clinically. We note that nearly all participants used multiple methods to give confidence to their results and should have used their uncertainty budget to indicate any issues arising from time constraints. One group managed to make three different measurements in the 2‐hour period.

Some of the detectors used in this comparison also may have not been used in such small fields before. All groups using active detectors used the ARPANSA‐supplied 3D water scanning tank to position their detectors in the 5 mm field. Scans in orthogonal directions were generally performed to find the center of the field; however, repeating these measurements was generally not possible due to time constraints. The water tank positioning reproducibility was tested after the measurements, and it was shown to reposition the detector to within the uncertainty quoted by the manufacturer. A few groups moved the detector in small increments near the center of the field to find the maximum response. Some participants who measured the output factor with Gafchromic EBT3 film approximated their usual procedures with the number of films irradiated at once, the total number of films irradiated, irradiation medium, and the measurement of dose linearity curves possibly being different to their usual procedures due to time limitations.

The correction factors used to correct the measurements of the various detectors in the 5 mm and 10 × 10 cm fields to an output factor are included in Table 3. The group who used the air core scintillation detector would have corrected their diode measurement based on the scintillation detector result which would have made these results identical. Thus, their diode result was reported without a correction factor with the assumption that it had over responded. For all other instances where the correction factor is one, it is presumed that the participant considered that a correction factor was not needed or they could not calculate or determine an accurate value for an appropriate correction factor. In the latter cases, we expect that the quoted uncertainty would include an estimate of the systemic uncertainty caused by not having a correction factor. The different sources of correction factors are also listed in Table 3. Different correction factors from separate publications have been applied to experimental results utilizing the same type of detector. For some measurements, the average of a range of published corrections has been used. The case of the correction factors used for the PTW 60019 microDiamond highlights some differences in the application of correction factors from the literature. Two of the three results used the same publication to obtain a correction factor, but the two values applied from this publication differed by 1.0%. The third result used a correction factor from a different publication which was 6.4% larger than the average of the other two correction factors. One publication based their correction factor on Monte Carlo calculations and the other on alanine measurements.^10^, ^16^ It is interesting to note that although such a range of correction factors were applied to these three results, the standard deviation was only 0.4%. Dieterich S et al. have measured cone factors with a number of diode detectors also used in this work allowing relative comparisons between their corrections to be made.^19^

Each participant was asked to provide the uncertainty in their output factor measurements including both random and systematic components. The uncertainty for each output factor measurement shown in Fig. 2 was calculated by the participant and is quoted at a confidence level of 95%. It was not modified in any way by the authors. For some results, it is possible that these were calculated as the Type A variation in the experimental measurement only and thus are underestimates. It was requested that each result is supplied with an expanded uncertainty containing all relevant Type A and Type B uncertainties. All uncertainty calculations provided here and in the clinic should include Type B uncertainties as outlined in the paper by Hill.^22^The standard deviation of all reported output factors was 5.6%. If the outlier ionization chamber result is excluded, the standard deviation reduces to 3.6%. This is still larger than the general recommendation on accuracy in radiotherapy of 3%.^23^ The results were also analyzed by dividing by the correction factors listed in Table 3 to investigate the influence of the stated correction factors. The standard deviation was then 8.4% (or 7.2% excluding the ionization chamber outlier result). The large increase is mainly due to the ion chamber results which require large correction factors. If these are excluded, the standard deviation is reduced to 3.8%. For comparison, the IAEA TRS‐398 estimate of the standard uncertainty in the calibration of a high‐energy photon beam in reference conditions is 1.5%.^1^

Nine of the 30 reported output factors were from detectors which had not been used to measure clinically employed small fields. If these output factors were excluded, then the standard deviation of the results increased from 3.6% to 3.9% indicating that these results did not contribute to an increase in the overall variability in the results. If the results only included those detectors which had been used to measure clinical fields equal to or smaller than that measured in this comparison, then the standard deviation remained unchanged at 3.6% also indicating no bias when only including those results.

## 5 CONCLUSION

A total of 15 independent groups travelled to ARPANSA in April 2016 to measure the output factor of a 5 mm cone on a nominal 6 MV linear accelerator photon beam. A wide range of detectors and methods were used to measure the output factor. The correction factors employed and general formalism of the measurements have been presented providing an indication of the current clinical work being done in this area. The standard deviation of all the results (excluding an outlier from an ionization chamber known to require a large correction) was 3.6%. The results give an indication of the consistency of the small‐field dosimetry being performed in Australia at the current time. Some care is needed in interpreting the results. In particular, time limitations and lack of a similar 5 mm field in every clinic meant that not all the facilities were attempting dosimetry with the same rigor. Several facilities failed to understand the accepted ways to report and/or calculate uncertainties, which resulted in a large variation in the uncertainty estimates. We expect that the publication of an accepted protocol for small‐field measurements would improve the consistency of the measurements and reporting of uncertainties that have been observed in this work.

## CONFLICT OF INTEREST

The authors have no conflicts of interest relevant to the content of this article.
